# DBscorer: An Open-Source Software for Automated Accurate Analysis of Rodent Behavior in Forced Swim Test and Tail Suspension Test

**DOI:** 10.1523/ENEURO.0305-21.2021

**Published:** 2021-11-02

**Authors:** Arnab Nandi, Garima Virmani, Aatmika Barve, Swananda Marathe

**Affiliations:** Centre for Neuroscience, Indian Institute of Science, Bangalore 560012, India

**Keywords:** behavioral analysis, depression, forced swim test, software, tail suspension test

## Abstract

Forced swim test (FST) and tail suspension test (TST) are commonly used behavioral tests for screening antidepressant drugs with a high predictive validity. These tests have also proved useful to assess the non-motor symptoms in the animal models of movement disorders such as Parkinson’s disease and Huntington’s disease. Manual analysis of FST and TST is a time-consuming exercise and has large observer-to-observer variability. Automation of behavioral analysis alleviates these concerns, but there are no easy-to-use open-source tools for such analysis. Here, we describe the development of Depression Behavior Scorer (DBscorer), an open-source program installable on Windows, with an intuitive graphical user interface (GUI), that helps in accurate quantification of immobility behavior in FST and TST from video analysis. Several calibration options allow customization of various parameters to suit the experimental requirements. Apart from the readout of time spent immobile, DBscorer also provides additional data and graphics of immobility/mobility states across time revealing the evolution of behavioral despair over the duration of the test and allows the analysis of additional parameters. Such comprehensive analysis allows a more nuanced understanding of the expression of behavioral despair in FST and TST. We believe that DBscorer would make analysis of behavior in FST and TST unbiased, automated and rapid, and hence prove to be helpful to the wider neuroscience community.

## Significance Statement

Forced swim test (FST) and tail suspension test (TST) are commonly used to rapidly screen novel antidepressant compounds. They are also used to assess the non-motor behaviors in models of Parkinson’s disease and Huntinton’s disease. However, the manual analysis of behavior is time-consuming and subject to observer-to-observer variability. The tools available for automation are either difficult to use, are expensive, require special apparatus, or not comprehensively validated. Depression Behavior Scorer (DBscorer), described here, is an open-source software for Windows with a user-friendly graphical user interface (GUI), which we have extensively validated against the performance of highly trained human scorers. We believe that the ease of installation and use, as well as the high accuracy would lead to a widespread adoption of DBscorer by the neuroscience community.

## Introduction

The serendipitous discovery of antidepressant drugs in the 1950s (Loomer et al., 1957; Kuhn, 1958) led to a quest to understand their mechanisms of action. This necessitated the development of suitable rodent models to study the action of antidepressant drugs. In the 1970s, Porsolt and colleagues described a new test to model behavioral despair in rodents (Porsolt et al., 1977, 1978). The test, called the forced swim test (FST), involves introducing the animal (mouse or rat) into a narrow cylindrical container containing water. After the initial period of vigorous swimming activity, the emergence of behavioral despair is evident from the time spent immobile. It was shown that a single injection with various classes of antidepressant drugs was sufficient to decrease the time spent immobile in the FST (Porsolt et al., 1977, 1978; Detke et al., 1997). Further, a dry version of FST, called the tail suspension test (TST), was proposed, where a mouse is suspended by its tail and the time spent immobile is scored as a measure of despair (Steru et al., 1985).

Both FST and TST have become classical tests for assessing depressive-like behavior in rodents. Since the initial study by Porsolt and colleagues, several other studies also reported that a single injection with antidepressant drugs significantly reduces the immobility in both FST and TST, allowing the test to be used to rapidly screen novel compounds for antidepressant-like activity (Castagné et al., 2009). Since these tests require the rodents to perform vigorous and coordinated motor activity under a stressful environment, these tests also find use in the fields of movement disorders (Pouladi et al., 2009; Campos et al., 2013; Bonito-Oliva et al., 2014; Du et al., 2015; Soylu-Kucharz et al., 2016).

However, these tests often suffer from observer-to-observer variability; and reproducibility is often a concern (Kara et al., 2018; Smalheiser et al., 2021; Trunnell and Carvalho, 2021). Moreover, the manual analysis of behavior is extremely time-consuming, thus hampering the utility of these tests for high throughput screening of candidate compounds.

Automation of the behavioral analysis in FST and TST could help in objective, quick and highly reproducible analysis of behavior. TST lends itself to easy automation through the use of a strain gauge to which a mouse is suspended by its tail and the changes in the force on the strain gauge is measured, which can then be used to distinguish mobility from immobility. This approach was first described in 1987 and multiple manufacturers now have strain gauge-based TST devices available for purchase (Steru et al., 1987; Mombereau et al., 2004; Cryan et al., 2005). Apart from the costs of acquisition and maintenance, a big disadvantage with this method is the number of mice that can be studied in parallel, which is dictated by the configuration of the device. On the other hand, the automation of FST is principally done through video analysis (Crowley et al., 2004; Gersner et al., 2009). Analysis of TST can also be done through video analysis, thus bypassing the need to use a specialized apparatus. Such analyses are often performed using commercially available software by tracking the frame-to-frame variations, changes in the position of the centroid, etc. (Juszczak et al., 2006). However, to our knowledge, there are no easy-to-use free open-source softwares available for FST and TST. Furthermore, an easy and intuitive graphical user interface (GUI) is only possible if the tool is specifically designed for FST and TST, since the general-purpose tools would not allow easy calibration of parameters that are relevant to FST and TST. Here, we describe the development of Depression Behavior Scorer (DBscorer), a MATLAB-based tool, designed for rapid and automated phenotyping of behavioral analysis in FST and TST. We validated the performance of the software against that of trained scorers and compared the behavior in an experiment involving a chronic mild unpredictable stress (CMUS) in mice. As long as the background provides sufficient contrast, DBscorer can be used on black as well as white animals. We believe that ease of installation and an intuitive GUI of DBscorer would help researchers with no programming knowledge to perform automated analysis of behavior in FST and TST and would thus help in a standardized, unbiased and objective analysis of behavior.

## Materials and Methods

### Animals

Six-month-old C57BL6/J male mice were used for experiments. Animals were bred and housed in the Indian Institute of Science and the experiments were performed in accordance with the protocols approved by the institutional animal ethics committee of Indian Institute of Science. Mice were housed in groups of three to five animals per cage and were maintained on a 12/12 h light/dark cycle with access to food and water *ad libitum*. Usage of animals was reduced as much as possible in accordance with the principle of 3Rs.

### FST

To quantify the behavioral despair in FST, the mice were allowed to swim in a 2L glass beaker filled up to 75% of its capacity with water. Before each test, the beaker was thoroughly cleaned. Water temperature was maintained between 21°C and 25°C. Mouse was placed in the middle of the container by gently holding its tail. Their behavior was videotaped for 5 min and the behavior over the entire 5 min period was analyzed. The FST protocol was adapted from previous studies (Castagné et al., 2011; Jaggar et al., 2017); 6-min-long FST protocols have also been used, with the first 2 min removed from analysis (Can et al., 2012a; Kara et al., 2018). The choice of the method in this study was based on a protocol standardized earlier in a similar chronic stress paradigm (Virmani et al., 2021). Every effort was made to minimize the effects of reflected light from the water surface. Lights were mounted on top of the behavioral setup at a height of 10 feet, with a light intensity of 100–120 lx at the level of the beakers. Non-reflective matte-finished white surface was placed behind the beakers to minimize the glare. The camera was set horizontally at water level and at a distance of three feet from the beakers. The position of the camera and beakers remained fixed over all the video recordings across multiple days.

After the test, mice were dried and put into a warm cage (30–33°C) for 20 min before returning to their home cage. For manual analysis, the mouse was considered immobile when floating passively and making only movements that were required to keep its head above water. Time spent immobile was reported as a % of total time.

### TST

For the TST, mice were hung by their tails for a period of 5 min. Since C57BL6/J mice show extensive tail climbing behavior as reported earlier (Can et al., 2012b), we passed the tail through a lightweight plastic tube (0.5 g) to prevent tail climbing as described previously (Can et al., 2012b). The lighting conditions and camera placement were similar to FST. After the tests were done, mice were returned temporarily to a holding cage until all the animals from their home cage were tested.

### CMUS

CMUS was performed as described earlier (Virmani et al., 2021). In brief, mice were subjected to one to two stressors per day for 21 consecutive days. Stressors were picked randomly from a set of stressors described earlier (Virmani et al., 2021) and were administered at a random time during the day. FST was performed on day 1 and day 19. TST was performed on days 2 and 17. These tests not only served as stressors but helped in the assessment of the emergence of behavioral despair as a result of CMUS.

### Video acquisition

The videos were acquired using a Nokia 6.1 mobile camera mounted horizontally at the level of the animal. The videos were converted to 15-fps .mp4 format using the ffmpeg tool (http://ffmpeg.org/). The lighting conditions as well as the white background were selected so as to provide the best possible contrast, while minimizing the glare.

### Development of DBscorer

Code for the DBscorer software was written in MATLAB 2020 (The MathWorks). Code can either be run on MATLAB or as a standalone program on Windows requiring MATLAB runtime 9.9 (The MathWorks). The user interface was built using the MATLAB App Designer. The interface is very intuitive and easy to use. The program analyzes video files in multiple video formats like .avi, .mp4, .mov, which are supported by MATLAB. It takes various user inputs like start and endpoints, area within the video to be analyzed, blurring and threshold values. Then it converts each frame to binary images according to user inputs. Threshold values were extensively tested and the recommended thresholds are given in Table 1.

**Table 1.** ROC analysis and optimum threshold values

TST time (s) removed	Area under the curve	Optimum threshold
0	0.9047	0.5806
1	0.9598	0.6506
2	0.9775	0.7407
3	0.9847	0.7808**
FST time (s) removed	Area under the curve	Optimum threshold
0	0.9154	2.2207
1	0.9600	2.2306
2	0.9694	2.2306
3	0.9735	2.5861**

To find the optimum Δ area thresholds for each test, ROC curves were plotted with 0–3 s removed around the behavioral state transitions. The table shows the area under the ROC curves and corresponding optimum thresholds. ** recommended Δ area threshold (%) for each test.

Difference between the area of the binarized image as a percentage of area of the previous frame is then calculated and averaged for each second. When this number is below a given threshold, the animal is considered to be immobile. The data are analyzed in blocks of user-defined time lengths, and immobility time is then calculated for each of these blocks.

For optional calibration or manual scoring of behavior, videos can be analyzed manually by the user using the software interface. It is recommended to use a video with approximately equal time of two states (mobility and immobility) to avoid any bias during calibration. The user has to press a toggle button when the animal becomes immobile to start quantification of immobility time, and then to press it every time when the animal transitions between the mobility and immobility states. For each video, an output file is created containing the timestamp for every second. Score of 1 is assigned to immobility (magenta toggle) and 0 to mobility (green toggle) for each second.

We have used 60% of all videos from each set to get the optimum threshold. The change in percentage area is then analyzed automatically by the software using various area threshold values and results for each second are systematically compared with the manual score to generate a receiver-operating characteristic (ROC) curve. We observed that humans typically take between 1 and 3 s to respond to the change of behavioral state with a key press, perhaps as a result of a combination of indecisiveness, inherent ambiguity in assessing an animal’s behavior and a lag in motor response. On the other hand, the behavioral state transitions are instantaneous in the case of automated analysis. Hence, the match between the two methods was poor around the boundaries between immobility and mobility states, but the match was stronger away from the boundaries. Since the time lags for a human scorer are not constant throughout, we could not artificially align the data by shifting the time-series. Hence, we removed 3 s around the boundaries while obtaining the optimum thresholds. The optimal threshold is determined from the ROC curve and reported in the user interface and in the output file. The formula is given below, where c is the cut-point: Optimum Cut − Point(c) = Sensitivity(c) × Specificity(c) (Unal, 2017).

We take the threshold with the maximum value of the optimal cut-point.

### Code accessibility

The source code is available as supplemental data (Extended Data 1) and in the online repository at http://github.com/swanandlab/DBscorer under a GPLv3 license. Periodic updates to this code, if any, will be made available on the link provided above.

10.1523/ENEURO.0305-20.2021.ed1 Extended Data 1 DBscorer Code and executables. Download Extended Data 1, ZIP file.

### Statistical analysis

All analyses were done in MATLAB (2020b) or GraphPad Prism. Pearson’s correlation and corresponding *p* values were calculated and the scatter plots with linear regression were made using GraphPad Prism. The Bland–Altman plot was plotted, and the bias was calculated using GraphPad Prism. The two group comparisons for the CMUS experiments were made using paired two-tailed Student’s *t* test and the data were plotted as bar graphs depicting mean ± SEM with overlaid scatter plot using GraphPad Prism. All other plots were prepared in MATLAB.

## Results

### Development of DBscorer and the GUI and the estimation of optimum threshold

**Figure 1.**
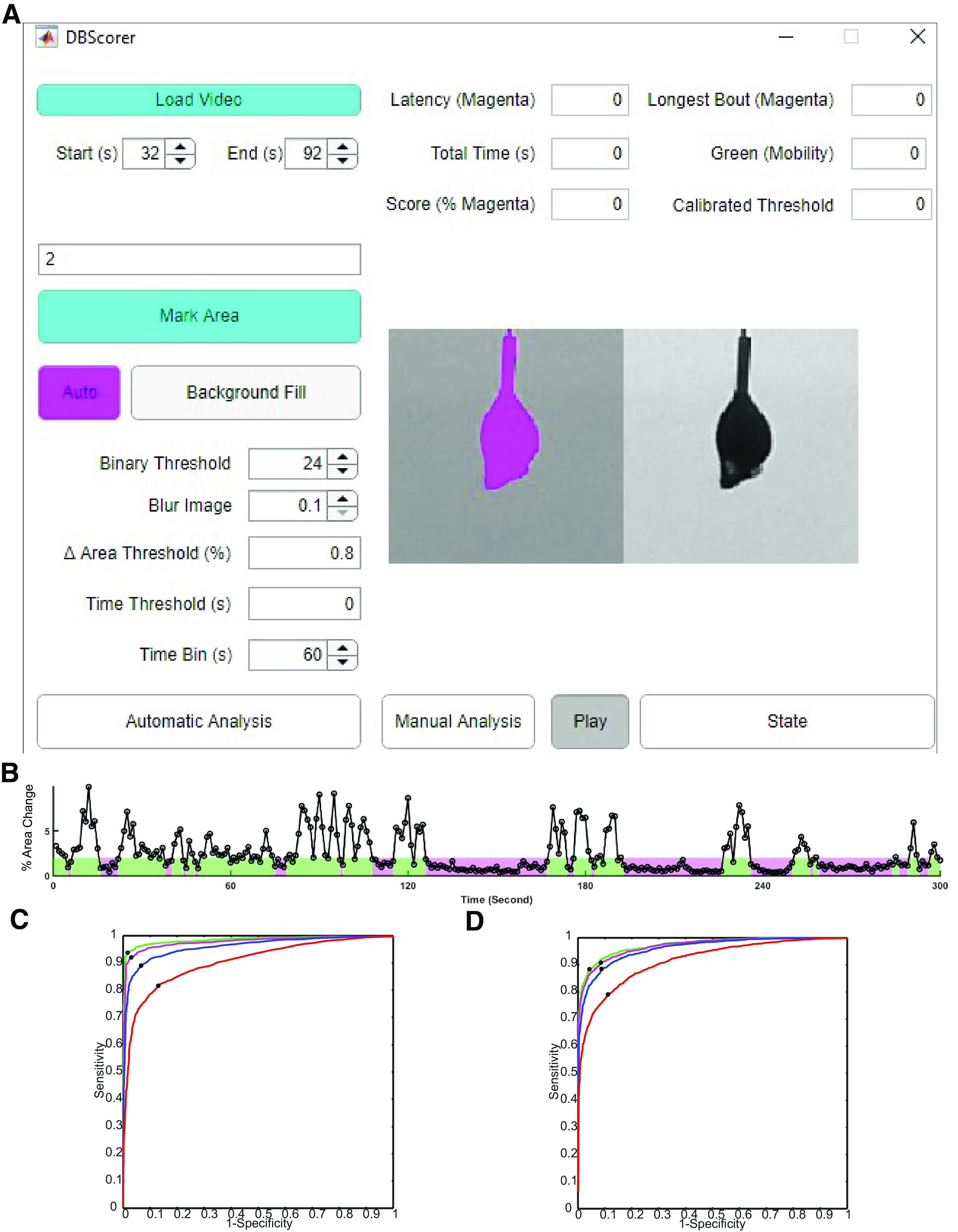
Development of DBscorer and the GUI and the estimation of optimum threshold. ***A***, Screenshot of the DBscorer user interface showing a TST video loaded for analysis. ***B***, The representative line plot shows the % change in the area as a function of time. Epochs of immobility are shown in magenta and epochs of mobility are depicted in green based on the optimized threshold. ROC curves for (***C***) TST and for (***D***) FST with 0 s (red), 1 s (blue), 2 s (magenta), or 3 s (green) removed from the behavioral state transitions. Sensitivity values at the optimum thresholds are shown by black dots for each ROC curve.

We developed DBscorer in MATLAB as described in the materials and methods. The GUI (Fig. 1*A*) was designed in MATLAB App Designer and the script was compiled as an executable file using MATLAB Compiler. This allows direct installation on Windows as a standalone program. A typical workflow (Fig. 1*A*; Movie 1) involves loading the video, selecting time boundaries of the part of the video that needs to be analyzed and marking the minimum area such that the animal remains within the selected area throughout the test. The area is marked using a multi-point selection, after which a rectangular area from the video is cropped out for analysis. This is followed by automated thresholding, though it is possible to input a user-defined binary threshold value. By pressing the “background fill” button and by marking the outer boundaries of the animal, we can estimate the background behind the animal. Following this, the analysis can be done completely automatically by pressing “automatic analysis.” DBscorer can also be used to perform the analysis manually by pressing the “manual analysis” button. For manual analysis, video playback can be controlled using the Play/Pause toggle switch. Another toggle switch called “state” is used to alternate between mobility and immobility during manual analysis.

Movie 1. Demonstration of installation and use of Dbscorer. The video shows how to install the appropriate version of the MATLAB runtime and DBscorer and how to perform the analysis automatically as well as manually. 10.1523/ENEURO.0305-21.2021.video.1

For automated analysis, we tested three different parameters for their correlation with human scorers. These parameters included changes in object length, object area and frame-by-frame variation. While change in object length was marginally better in the case of TST, change in area was more versatile and suited for both FST as well as TST. Hence, change in the area of the object was used for further validation (Fig. 1*B*). Δ area threshold as a % of the area of the previous second is used to classify the data into mobility or immobility (Fig. 1*B*). Furthermore, manual analysis can be done to automatically calibrate the Δ area threshold (%). Table 1 summarizes the Δ area thresholds from our calibrations, which we recommend to the users.

We used the ROC curves to obtain an optimum threshold for each test as described in the materials and methods (Fig. 1*C*,*D*; Table 1). Using these thresholds, we sought to compare the performance of DBscorer and human scorers in FST and TST.

### Comparison of automated analysis with DBscorer against manual analysis in TST

**Figure 2.**
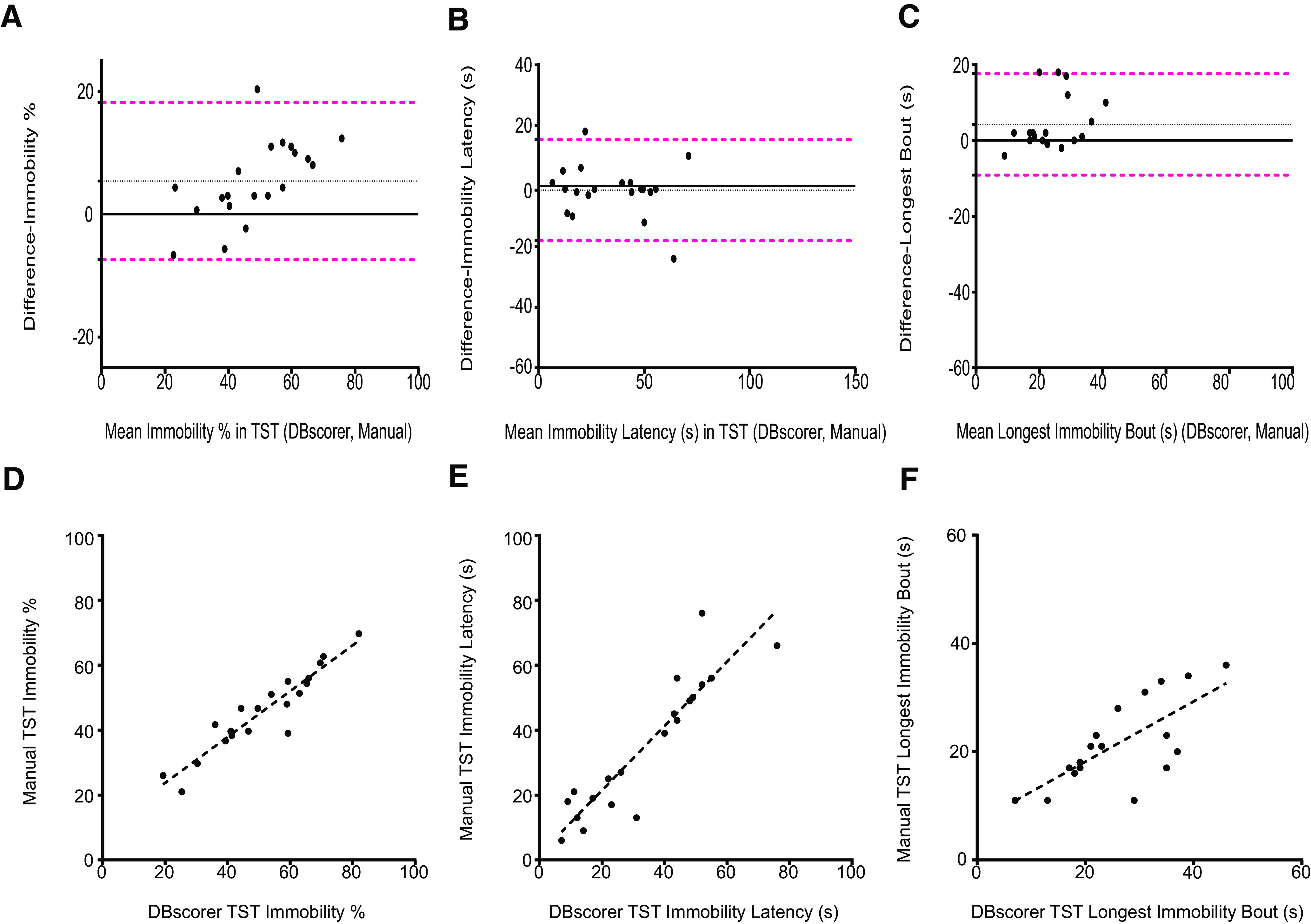
Comparison of automated analysis with DBscorer against manual analysis in the TST. Bland–Altman plots for TST are plotted using the data obtained with DBscorer and manual analysis for (***A***) % time spent immobile, (***B***) latency to the first bout of immobility (seconds), and (***C***) the duration of the longest bout of immobility (seconds). Black solid line at zero denotes complete agreement between the two methods. Dotted black line denotes the mean. Dotted magenta lines depict 1.96 SDs (95% limits of agreement) away from the mean. Scatter plots with an overlaid linear fit showing the correlation between the manually obtained data with the data obtained from DBscorer for (***D***) % time spent immobile, (***E***) latency to the first bout of immobility (seconds), and (***F***) the duration of the longest bout of immobility (seconds). Pearson’s correlation coefficient and *p* value were used to determine the correlation; *n* = 20 videos.

To validate the performance of the DBscorer, comparisons with the manual analysis were made for both TST as well as FST. For TST, we used 20 videos from C57BL6/J mice subjected to TST. We computed three parameters for each mouse, namely, percent time spent immobile, latency to the first bout of immobility, and the longest bout of mobility. Although the traditional method of manual analysis using a stopwatch does not allow the measurements of parameters other than the time spent immobile, manual analysis on DBscorer allows these measurements. We used the Bland–Altman plots to assess the agreement between the two methods for all three parameters. We found a good agreement between DBscorer and manual scoring on all three parameters for TST (Fig. 2*A–C*). The bias for the immobility % was 5.4 and 95% limits of agreement from −7.40 to 18.20 (Fig. 2*A*). For latency to immobility, the bias was −1.35 the 95% limits of agreement were −18.05 to 15.35 (Fig. 2*B*). In the case of the longest bout, the bias was 4.25, and the 95% limits of agreement were from −9.13 to 17.63.

We next assessed the correlation between the results obtained using the two methods. In the percent time spent immobile, we found a strong correlation between DBscorer and manual analysis [*R*^2^ (20) = 0.87, *p* < 0.0001; Fig. 2*D*]. The correlation coefficients and *p* values for latency to immobility were *R*^2^ (20) = 0.83 and *p* < 0.0001, respectively (Fig. 2*E*), while those for the longest bout were *R*^2^ (20) = 0.52 and *p* < 0.0003, respectively (Fig. 2*F*).

### Comparison of automated analysis with DBscorer against manual analysis in FST

**Figure 3.**
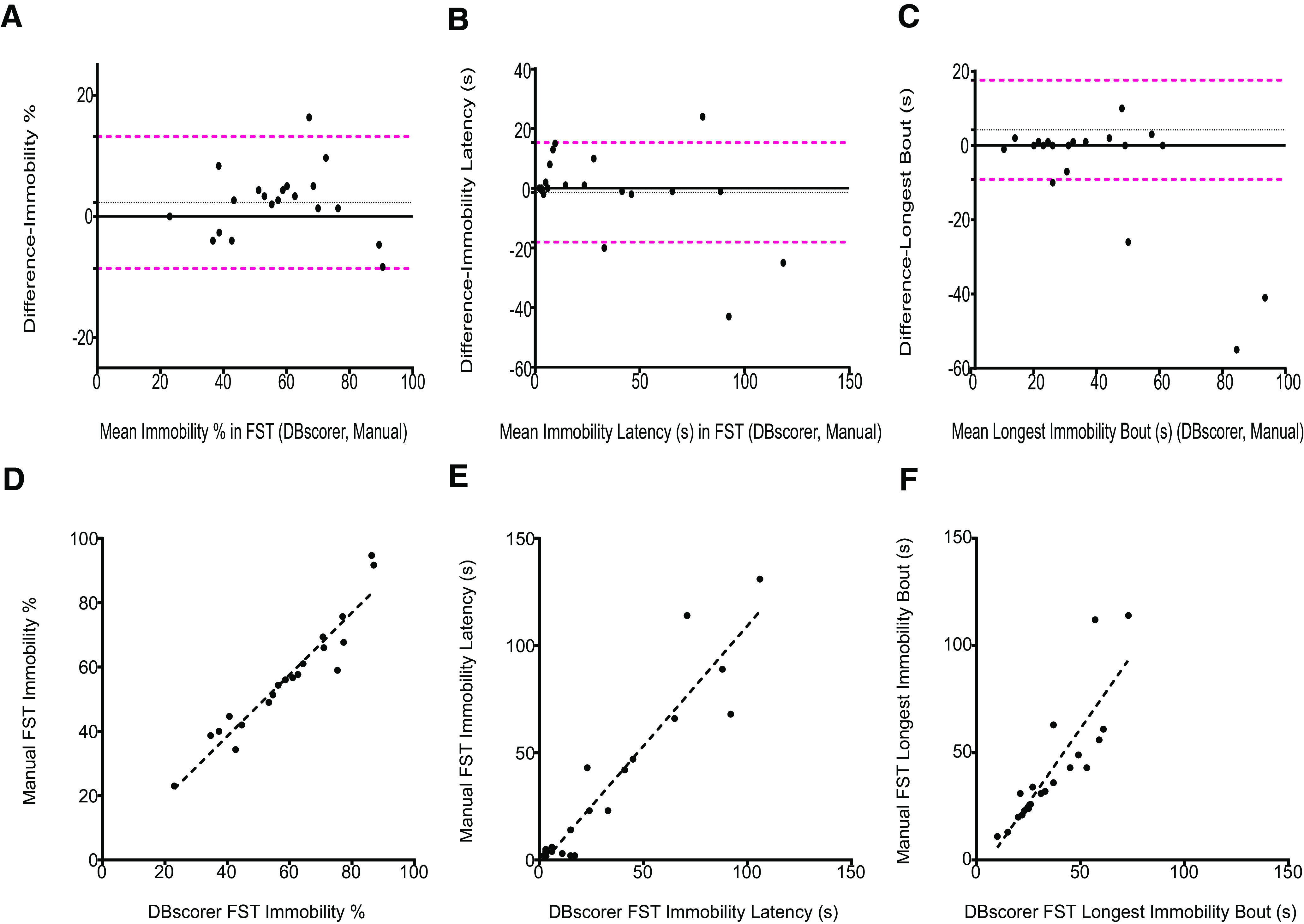
Comparison of automated analysis with DBscorer against manual analysis in the FST. Bland–Altman plots for FST are plotted using the data obtained from DBscorer and with manual analysis for (***A***) % time spent immobile, (***B***) latency to the first bout of immobility (seconds), and (***C***) the duration of the longest bout of immobility (seconds). Black solid line at zero depicts complete agreement between the two methods. Dotted black line denotes the mean. Dotted magenta lines depict 1.96 SDs (95% limits of agreement) away from the mean. Scatter plots with an overlaid linear fit showing the correlation between the manually obtained data with the data obtained from DBscorer for (***D***) % time spent immobile, (***E***) latency to the first bout of immobility (seconds), and (***F***) the duration of the longest bout of immobility (seconds). Pearson’s correlation coefficient and *p* value were used to determine the correlation; *n* = 20 videos.

For FST, we used 20 videos from C57BL6/J mice subjected to FST. We found a good agreement between DBscorer and manual scoring on all three parameters for FST (Fig. 3*A–C*). The bias for % immobility was 2.3 and 95% limits of agreement were from −8.58 to 13.18 (Fig. 3*A*). For latency to immobility, the bias was −1.10 the 95% limits of agreement were −29.55 to 27.35 (Fig. 3*B*). In the case of the longest bout, the bias was −5.95, and the 95% limits of agreement were from −37.57 to 25.67 (Fig. 3*C*).

We next assessed the correlation between the results obtained using the two methods. In the percent time spent immobile, we found a strong correlation between DBscorer and manual analysis [*R*^2^ (20) = 0.90, *p* < 0.0001; Fig. 3*D*]. The correlation coefficients and *p* values for latency to immobility were *R*^2^ (20) = 0.87 and *p* < 0.0001, respectively (Fig. 3*E*), while those for the longest bout were *R*^2^ (20) = 0.73 and *p* < 0.0003, respectively (Fig. 3*F*).

### Analysis of TST and FST behavior in the CMUS paradigm

**Figure 4.**
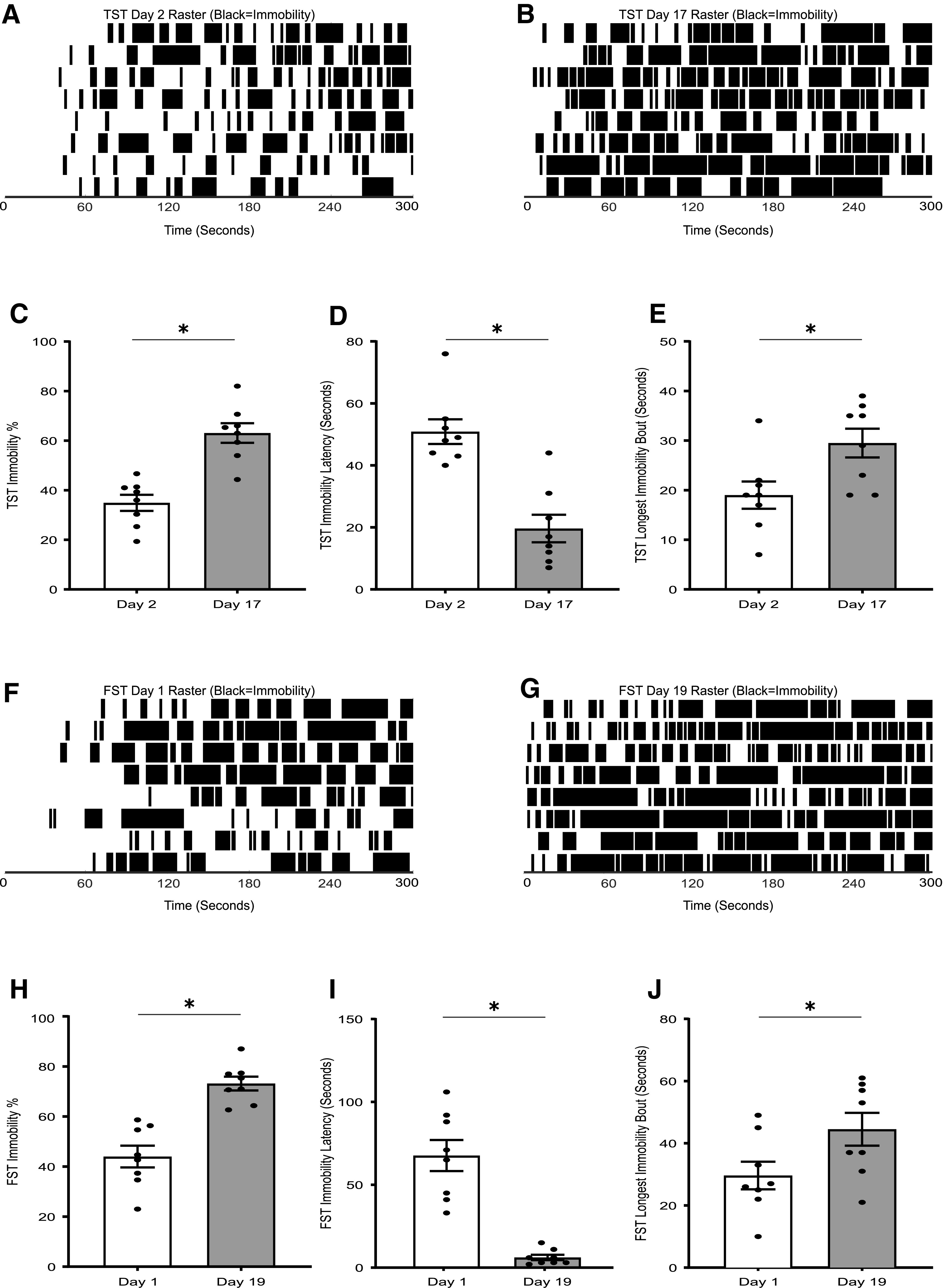
Analysis of TST and FST behavior in the CMUS paradigm. Raster plots show the bouts of immobility (black) against time in the TST at (***A***) day 2 as compared with (***B***) day 17 of the 21-d-long CMUS paradigm. Bar graphs comparing the behavior in TST at day 2 and day 17 show (***C***) a significant increase in the % of time spent immobile, (***D***) a significant decrease in the latency to the first bout of immobility (seconds), and (***F***) a significant increase in the duration of the longest bout of immobility (seconds). Raster plots reveal the bouts of immobility (shown in black) as a function of time in the FST at (***F***) day 1 and (***G***) day 19 of the 21-d-long CMUS paradigm. Bar graphs show (***H***) a significant increase in the % of time spent immobile, (***I***) a significant decrease in the latency to the first bout of immobility (seconds), and (***J***) a significant increase in the duration of the longest bout of immobility (seconds) in the FST paradigm shows; *p < 0.05, two-tailed paired *t* test; *n* = 8 mice per group.

To test DBscorer in a real-world experiment, we used a 21-d CMUS paradigm. It was shown that such a paradigm leads to a significant increase in immobility and other related parameters (Virmani et al., 2021). TST was a part of the CMUS paradigm as mentioned in the materials and methods section and was performed on day 2 and day 17 of the paradigm. In agreement with previously reported data, there was a significant increase in immobility at day 17 as compared with day 2 as seen from the raster plots of immobility (Fig. 4*A*,*B*). Comparisons using paired *t* tests also showed a statistically significant increase in the % time spent immobile (day 2: 34.92 ± 3.25%, day 17: 63.08 ± 3.95%; *p* = 0.0015; Fig. 4*C*), a decrease in the latency to immobility (day 2: 50.88 ± 3.98 s, day 17: 19.63 ± 4.44 s; *p* = 0.0002; Fig. 4*D*) and an increase in the length of the longest bout of immobility (day 2: 19.00 ± 2.74 s, day 17: 29.50 ± 2.89 s; *p* = 0.0221; Fig. 4*E*).

Time spent immobile in FST was analyzed on day 1 and day 19 of the CMUS paradigm, which showed an increase at day 19 as compared with day 1 as can be seen from the raster plots for FST (Fig. 4*F*,*G*). Paired *t* test analyses also showed a significant increase in time spent immobile (day 1: 44.00 ± 4.34%, day 19: 73.17 ± 2.76%; *p* = 0.0013; Fig. 4*H*), a decrease in the latency to immobility (day 1: 67.63 ± 9.37 s, day 19: 6.12 ± 1.63 s; *p* = 0.0003; Fig. 4*I*) and a significant increase in the duration of the longest bout of immobility (day 1: 29.63 ± 4.44 s, day 19: 44.50 ± 5.27 s; *p* = 0.0232; Fig. 4*J*).

Taken together, the automated analysis on DBscorer revealed the emergence of the depressive-like behavior in a CMUS paradigm not just with the conventional parameter of the % of time spent immobile, but also with additional parameters and a raster plot. We believe that the DBscorer would prove to be a faster and more objective analysis method that will accelerate the screening for novel antidepressant compounds.

## Discussion

Shortcomings of currently prescribed antidepressants have necessitated the search for novel antidepressant drugs. This search requires screening methods that are easy, efficient, and objective. FST and TST provide a quick behavioral test for screening novel compounds for their antidepressant-like activity. The analysis of behavioral despair in these tests typically involve the quantitation of the time that the rodents spend immobile. While manual analysis by a trained scorer is a norm, such manual analyses are often tedious, inefficient and subject to high interscorer variability. Fortunately, these methods do lend themselves to automation based on video analysis. Automated detection of the rodents in the video frame followed by measurements of the changes in various features of the object between frames has been tried and published before (Hédou et al., 2001; Kurtuncu et al., 2005; Rocha et al., 2005; Cryns et al., 2007; Juszczak et al., 2008; Yuman et al., 2008; Kulikov et al., 2010; Hayashi et al., 2011; Gao et al., 2014; Pennington et al., 2019; Sturman et al., 2020). TST has also been automated using a strain-gauge apparatus (Steru et al., 1987; Crowley et al., 2004; Mombereau et al., 2004; Strekalova et al., 2004; Cryan et al., 2005; Alexandre et al., 2006). But video analysis is preferred since it does not require any special apparatus, and the number of animals that can be simultaneously tested is not dictated by the configuration of the apparatus (Can et al., 2012b).

To our knowledge, there is a dearth of video analysis tools that are user friendly with a GUI, free-to-use and extensively validated. Here, we describe the development of DBscorer, an open-source software written in MATLAB, and has an intuitive GUI for ease of use. We tested the performance of DBscorer against that of experienced scorers. We found a significant correlation between manually scored data and the data obtained from DBscorer (Figs. 2, 3). Furthermore, we also used DBscorer to analyze a real-world experiment to monitor behavioral changes as a result of a 21-d CMUS paradigm. In addition to the parameters that can be calculated manually, DBscorer returned additional parameters as well as a raster plot depicting the evolution of behavioral despair as a function of time (Fig. 4). These parameters and graphics can also be generated using event recording tools such as ETHOM (Shih and Mok, 2000), but this needs a completely manual analysis of behavior. We believe that the detailed behavioral analysis by DBscorer can provide additional insights into the effects of experimental interventions.

When an animal is first introduced into the water during FST, or suspended by its tail for TST, they typically exhibit erratic mobility behavior for some initial duration that can obscure the real effects of the treatment being studied. Hence, typically the initial period is removed from the analysis. Every lab has its standard operating procedure, and anywhere from 0 to 2 initial minutes are removed from the analysis. We believe that this would not only depend on the species and the strain of animals being tested but may also vary depending on the specific experimental intervention being studied. The raster plot provided by DBscorer would help make a more informed and objective decision on the specific period to analyze from the total length of the test. In addition, other parameters such as latency to immobility and the duration of the longest bout could also provide additional insights into the animals’ responses to the experimental interventions. Furthermore, the source code for DBscorer has been made open source so that the wider community can collaboratively improve on the analysis and add additional features that may further our understanding of behavioral despair in rodents.

We believe that the availability on the Windows platform and an intuitive GUI would help DBscorer to be easily adopted by users with no knowledge of computer programming. On the other hand, more advanced users can modify and adapt the software as per their requirements. Moreover, continued collaborative development of DBscorer would help further improve the software. In summary, we believe that DBscorer would prove to be incredibly useful for the scientific community working on depression and antidepressant treatments in rodent models.

## Synthesis

Reviewing Editor: Christie Fowler, University of California, Irvine Decisions are customarily a result of the Reviewing Editor and the peer reviewers coming together and discussing their recommendations until a consensus is reached. When revisions are invited, a fact-based synthesis statement explaining their decision and outlining what is needed to prepare a revision will be listed below. The following reviewer(s) agreed to reveal their identity: Jill Turner.

Your manuscript was reviewed by two experts in the field. Both reviewers felt the manuscript provided important information for the field by automating the analysis of two commonly used assays for behaviors in rodents using an open-source, Matlab-based pipeline. However, it was felt that several revisions would strengthen the findings, which need to be addressed in a revision:

Major:

1. The manuscript title and text should be revised to decrease the emphasis placed on the relevance of the TST and FST in measuring depression. There is a significant controversy in the field as to whether these tests are valid measures of depression in animal models (see PMID: 31048727).

2. The introduction could pull in a wider audience by citing other fields that also use the tail suspension test and the forced swim test in behavioral analysis.

3. The FST section of the methods needs citations and probably a discussion of any differences in methodologies across lab. Is this a typical recording period?

4. The video acquisition period of the methods - giving the reader the background lighting conditions explicitly would greatly enhance reproducibility.

5. The justification for using 60% of all videos from each set to get the optimum threshold should be further explained.

6. There are other options for manual analysis of these tests, including free software like ETHOM, which would allow measurement of parameters like latency to the first bout and the longest bout etc.

7. Why was there no control group for the CMUS experiments that did not undergo the stress paradigm? What is the effect of repeated testing?

Minor:

1. In the first paragraph of the introduction, the authors state that rodents have proven to be invaluable animal models for studying the mechanisms of MDD pathophysiology and then that it remains challenging to model the core symptoms of MDD in lab animals. These two sentences feel in heavy conflict and if the authors could expand upon these points, it would enhance readability.

2. “However, these tests often suffer from observer-to-observer variability; and reproducibility is often a concern.” - this needs a citation

3. For the purposes of in-depth analyses - does this platform offer an easy to use binning approach?

## Author Response

On behalf of all authors, I would like to thank the anonymous reviewers and the editor for reviewing our manuscript. This is our point-by-point response to the concerns and suggestions of reviewers.

Synthesis of Reviews:

Significance Statement Comments for Author (Required):

The statement should be rephrased to decrease the emphasis on depression. Specifically, there is debate in the field as to the relevance of the FST and TST assessments as a measure of depression in rodent models.

We have de-emphasized the utility of these tests in depression and have also included additional applications of the tests from other fields.

Comments on the Visual Abstract for Author (Required):

N/A

Synthesis Statement for Author (Required):

Your manuscript was reviewed by two experts in the field. Both reviewers felt the manuscript provided important information for the field by automating the analysis of two commonly used assays for behaviors in rodents using an open-source, Matlab-based pipeline. However, it was felt that several revisions would strengthen the findings, which need to be addressed in a revision:

Major:

1. The manuscript title and text should be revised to decrease the emphasis placed on the relevance of the TST and FST in measuring depression. There is a significant controversy in the field as to whether these tests are valid measures of depression in animal models (see PMID: 31048727).

We have changed the title and removed the reference to depressive-like behavior. We have reduced the emphasis on “measuring depression” throughout the manuscript. We have also included the examples of the use of these tests in other fields (mainly movement disorders) in the abstract, significance statement, introduction and discussion.

2. The introduction could pull in a wider audience by citing other fields that also use the tail suspension test and the forced swim test in behavioral analysis.

We have made the suggested changes to the introduction.

3. The FST section of the methods needs citations and probably a discussion of any differences in methodologies across lab. Is this a typical recording period?

We have added citations for the FST method, different recording periods reported in the literature and the rationale for selecting the protocol used in the study.

4. The video acquisition period of the methods - giving the reader the background lighting conditions explicitly would greatly enhance reproducibility.

Details of video acquisition and lighting conditions have now been explained in detail in the materials and methods section.

5. The justification for using 60% of all videos from each set to get the optimum threshold should be further explained.

We split the available dataset into a training dataset (60%) and testing dataset (40%). 60-40 split was deemed to be the optimum compromise to calibrate the threshold and test its accuracy on the testing dataset. The training datasets were not used for testing.

6. There are other options for manual analysis of these tests, including free software like ETHOM, which would allow measurement of parameters like latency to the first bout and the longest bout etc.

We have added text highlighting this point and have cited the original ETHOM paper.

7. Why was there no control group for the CMUS experiments that did not undergo the stress paradigm? What is the effect of repeated testing?

In the CMUS experiment, behavior was compared between the first test done before the stress paradigm and the last test done at the end of the paradigm, using a paired t-test analysis. The purpose was just to show the data from a real-world experiment analyzed using DBscorer. The data obtained from DBscorer correlated strongly with manual analysis. There was no separate control group used in this experiment - the behavior data before starting the CMUS paradigm served as control (baseline). The experiment was designed to minimize animal usage for the purpose of software validation, while operating within the COVID restrictions on working hours.

We have not performed repeated testing for this particular experiment. However, the comparisons between manual and automated analysis (Figure 2 and Figure 3) have been performed on a variety of videos under different experimental conditions used in our lab for different projects. The strong correlation between the manual and automated data would hold for repeated tests as well, thus not hampering the utility of DBscorer for analysing the results of repeated testing.

Minor:

1. In the first paragraph of the introduction, the authors state that rodents have proven to be invaluable animal models for studying the mechanisms of MDD pathophysiology and then that it remains challenging to model the core symptoms of MDD in lab animals. These two sentences feel in heavy conflict and if the authors could expand upon these points, it would enhance readability.

In response to earlier suggestions, this part of the introduction has been removed altogether to reduce the emphasis on depression. Hence, this is no longer an issue.

2. “However, these tests often suffer from observer-to-observer variability; and reproducibility is often a concern.” - this needs a citation

We have added citations as suggested by the reviewer to strengthen this point.

3. For the purposes of in-depth analyses - does this platform offer an easy to use binning approach?

Binning is completely user-customizable using the input field “Time Bin (s)” in the GUI.
